# The burden of flashes and floaters in traditional general emergency services and utilization of ophthalmology on-call consultation: a cross-sectional study

**DOI:** 10.1186/s12886-022-02613-6

**Published:** 2022-10-04

**Authors:** Carl Shen, Alicia Liu, Forough Farrokhyar, Mark Fava

**Affiliations:** 1 Division of Ophthalmology, Department of Surgery, McMaster University, Hamilton, ON Canada; 2 Faculty of Health Sciences, McMaster University, Hamilton, ON Canada; 3 Departments of Surgery and Health Research Methods, Evidence, and Impact, McMaster University, Hamilton, ON Canada

**Keywords:** Flashes, Floaters, Triage, Consultation, Health Systems, Care Pathways

## Abstract

**Purpose:**

To characterize the healthcare utilization and clinical characteristics of patients presenting with flashes and/or floaters (F/F) in general emergency service (GES) settings.

**Methods:**

All adults presenting to GESs (emergency departments (EDs) and urgent care centers (UCCs)) with symptoms of F/F in Hamilton, Ontario between Jan. 1 – Dec. 31, 2018 were reviewed. Primary outcome was the proportion of patients presenting to GESs with F/F for which ophthalmology emergency services (OESs) were consulted. Secondary outcomes included features predictive of OES consultation by logistic regression and cost of GES utilization.

**Results:**

Of 6590 primary eye-related visits to GESs, 10.4% (687) involved symptoms of F/F. Mean age of patients with F/F was 57 ± 15 years, and 61% were female. Consultation rate to OESs for F/F presentations was 89% (608/687). Logistic regression identified symptoms ≤ 2 weeks (OR 8.0; 95% CI 2.3–28), ≥ 45 years age (OR 2.4; 95% CI 1.4–4.3), UCC setting (OR 2.7; 95% CI 1.6–4.6), headache (OR 0.22; 95% CI 0.12–0.41), and neurologic symptoms (OR 0.1; 95% CI 0.19–0.49) as variables predictive of OES consultation. Mean time from triage to discharge in GESs for F/F patients was 2.43 ± 2.36 h. Mean cost per visit was $139.11 ± $113.93 Canadian dollars. Patients for which OES were consulted waited a total of 1345 h in GESs and accounted for $81,879.70 in costs.

**Conclusion:**

Patients presenting with F/F in GESs consume considerable resources in healthcare expenditure and time spent in GESs and most receive OES consultation. Identifying these patients at triage may allow for increased efficiency for the healthcare system and patients.

**Supplementary Information:**

The online version contains supplementary material available at 10.1186/s12886-022-02613-6.

## Introduction

A common consult to ophthalmology emergency services (OESs) is the acute onset of flashes of light and new floaters (F/F) in an adult[1]. Patients with this complaint require a dilated fundus examination to rule out the presence of retinal tears or detachment. Often these patients present to general emergency services (GESs)[2] as a point of first contact with the healthcare system before being referred to OESs where a thorough posterior segment examination can be conducted.

Within North America, there is a trend towards increased utilization of GESs including emergency departments (EDs) and urgent care centers (UCCs)[3]. Eye-related complaints represent a substantial subset of visits in GESs, accounting for approximately 2 million visits to the ED in the United States every year[4]. These visits contribute to the overcrowding and overburdening of GESs which have been associated with poorer clinical outcomes[5]. In addition, several studies have demonstrated the increased cost of care delivered in a GES setting compared to an ambulatory clinic setting for the same complaint[6, 7]. There is considerable motivation to identify inefficiencies with the delivery of emergency eye care through current GESs where patients are and will continue to present. This point has been accentuated with the recent global SARS-CoV-2 pandemic as hospitals have been faced with the need to create bed space, minimize transmissibility, and patient and physician exposure, all while continuing to provide appropriate care[8]. One area that has been identified as a target for quality improvement in GESs is the optimization of triage and consult services[9]. GESs constitute approximately 50% of the utilization of OESs in Ontario[2] and represent a key first point of contact in the delivery of emergency eye care for many patients. Through the process of triage, the opportunity exists to both efficiently and safely identifying patients that might benefit from an expediate or alternative pathway of care.

The population of patients presenting with F/F in GESs has not been previously studied and may be particularly amenable to optimization of their care pathway upon first contact with the healthcare system.

The purpose of this study was to describe the healthcare utilization of patients presenting with F/F to GESs in a publicly funded health care system and secondarily characterize their demographic and clinical features, and identify features related to consultation of OESs. Our hypothesis was that a substantial portion of these patients would require further consultation from OESs and identification of these patients at their GES triage encounter would represent a potential target for optimizing delivery of care.

## Methods

### Study design and setting

This study was an institutional retrospective cross-sectional study. All patients presenting with symptoms of F/F to GESs between January 1st, 2018 – December 31st, 2018 were identified in a database of all GES primary eye-related complaints in the 2018 calendar year in Hamilton, Ontario, Canada. The study was approved by the institutional review board of the Hamilton Integrated Research Ethics Board #5388. The need for informed consent was waived by the Hamilton Integrated Research Ethics Board.

This database had been created through identification of emergency encounters where the primary complaint was eye-related as determined by the Canadian Emergency Department Information Systems (CEDIS) triage classification codes 501–511. Manual coding from free triage text to dichotomous presence of symptoms was previously performed using selected keywords and criteria (Supplemental Materials). In 2018 the city of Hamilton had an estimated population of 568,000. Its GES needs are served by two healthcare systems, Hamilton Health Sciences (HHS) and St. Joseph’s Healthcare Hamilton (SJHH). The HHS system consists of 3 academic teaching hospital EDs, 1 community hospital ED, and 1 UCC, and the SJHH system of 1 academic teaching hospital ED and 1 UCC. EDs in the city are accessible 24 h a day, 7 days a week with triage and assessment by an emergency physician. EDs are associated with hospitals with inpatient facilities and are intended for higher acuity patients. UCCs in the city are accessible 7 days a week between 8 am and 10 pm and are not associated with inpatient facilities. Triage is performed by registered nurses, but patients are similarly assessed by an emergency physician and are intended for less acute patients. In both settings, patients are registered, triaged and their vitals assessed, then seen by a physician. OESs in Hamilton are accessible by any health care provider through an on-call service 24 h a day, 7 days a week, for consultation within 24 h. Patients cannot contact the OESs directly. The OESs in Hamilton are staffed by ophthalmology residents/registrars and a staff consultant. The majority of OES patients are transferred to and assessed at the ambulatory care center where the university affiliated eye clinic is based, or to OES physician’s private offices. If the patient is non-transferable from the GES setting the OES physician will present to the GES however there is no OES physician normally based in the GESs.

Inclusion criteria for the study was any adult over the age of 18 presenting with a primary eye-complaint to a GES in the city of Hamilton in 2018, with triage symptoms containing F/F of any laterality. Exclusion criteria included any encounters where the patient left without being seen after triage, encounters where patients presented for the express purpose of imaging or medication administration as an extension of another encounter, follow-up visits arranged by emergency physicians, and inappropriately coded encounters that were not primarily eye-related.

Extracted fields included age, gender, site of presentation, date of admission, date of discharge, time of arrival, time of triage, time of discharge, Canadian Triage and Acuity Scale score (CTAS), CEDIS triage code, coded symptoms at triage, consultation to ophthalmology emergency services, recent (≤ 6 weeks) invasive ophthalmic intervention (surgery, injection, or laser procedure), acuity (acute ≤ 2 weeks, sub-acute > 2 and < 4 weeks, chronic ≥ 4 weeks), directed to GES by healthcare provider, and case costs for each encounter. Data collection was performed using standardized tables. Case costing data was provided by respective health care systems case costing departments and is presented in Canadian dollars ($) at 2018 values. Case costs were totals for each patient encounter including all staffing, material, medication, and investigation costs. Billing for the encounter by the emergency physician, which is to the province directly, was not included in this value.

The primary outcome measure was the proportion of patients for which OESs were consulted. Consultation to OES was defined as a direct communication to the ophthalmology on-call service (“paging”) for a patient to be seen within 24 h. GES encounters where the patient was directed to follow-up with their own ophthalmologist or optometrist, a non-urgent referral to an ophthalmologist was made, or where the patient was directed to obtain a referral to ophthalmology from another healthcare provider were not considered consultation of OESs. Additional outcome measures included factors related to consultation of OESs as determined by multivariable logistic regression.

### Statistical analysis

Consultation rate to OES was calculated as the number of encounters of F/F referred to ophthalmology on-call divided by the total number of F/F encounters that presented to GESs. One-step method multivariable logistic regression was performed to determine variables predictive of consultation of OESs. Variables that were included in the model were determined based on clinical plausibility and symptoms previously identified to be associated with the presence of retinal tear/detachment including, age ≥ 45,[10] ED vs. UCC site, acuity (chronic/sub-acute/acute), recent invasive ophthalmic intervention defined as laser, peri/intra-ocular injection, or surgery, directed to GES by healthcare provider, presence of subjectively decreased vision as a symptom[11], presence of visual field defect as a symptom[11], presence of neurologic symptoms, and presence of headache as a symptom. Odds ratio with the corresponding confidence intervals (CI) and p-values are reported. Statistical significance was considered for p values < 0.05. Statistical analysis was performed using SPSS version 20 (IBM Corporation, Armonk, NY).

### Ethics approval

Institutional review board approval was obtained from the Hamilton Integrated Research Ethics Board. The research adhered to the tenants of the Declaration of Helsinki.

## Results

### Characteristics of study subjects

In 2018, after exclusion criteria were applied, there were 6590 primary eye-related visits to GESs for adult patients. Of these visits, 687 (10.4%) involved symptoms of F/F (Table 1). 75% (513/687), presented in the UCC setting. Distribution of patients presenting across day of the week and month were similar throughout the study period. Mean age of F/F patients was 57.5 ± 15 years (range 18–95), and the majority were female (61%). The most commonly assigned CTAS score which was assigned for 322/687 (49%) F/F encounters was CTAS level 3 which are considered urgent conditions that could potentially progress to a serious problem requiring emergency intervention.

**Table 1:** Characteristics of Adult Patients Presenting with Flashes and/or Floaters to General Emergency Service Settings

Patient characteristics	
No. of patients (n)	687
Median age (years ± SD)	57.5 ± 15
Gender (female:male)	418:269
**Presentation characteristics (n[%])**	
Healthcare Setting	
Emergency department	174 [25%]
Urgent care center	513 [75%]
Canadian Triage Acuity Score	
1	0 [0%]
2	246 [36%]
3	322 [47%]
4	109 [16%]
5	9 [1.3%]
9	1 [0.1%]
Time of day	
Working hours (8:00–15:59)	522 [76%]
After hours (16:00–7:59)	155 [24%]
Weekday	
Sun	82 [12%]
Mon	109 [16%]
Tues	89 [13%]
Wed	94 [14%]
Thurs	98 [14%]
Fri	116 [17%]
Sat	99 [14%]
Season	
Jan – Mar	170 [25%]
Apr – Jun	165 [24%]
Jul – Sept	168 [24%]
Oct – Dec	184 [27%]

### Clinical features

The clinical features of the F/F patients are summarized in Table 2. Overall, 305/687 (44%) patients presented with floaters without flashes, 139/687 (20%) presented with flashes without floaters, and 243/687 (35%) presented with both flashes and floaters. Symptoms of flashes were unilateral in 347/382 (91%) of cases and symptoms of floaters were unilateral in 524/548 (96%) of cases. The most common associated ocular symptoms were decreased vision (24.8%), pain (11%), visual field defect (5.7%) and the most commonly associated systemic symptom was headache (10.2%) and all other systemic symptoms were < 2% (Supplemental Materials). 95% (653/687) patients presented with symptoms ≤ 2 weeks duration and 31/687 (4.5%) presented within 6 weeks of invasive ophthalmic intervention.

**Table 2:** Clinical Features of Adult Patients Presenting with Flashes and/or Floaters to General Emergency Service Settings

Symptom characteristic	
Flashes AND Floaters	243/687 [35%]
Floaters NOT Flashes	305/687 [44%]
Flashes NOT Floaters	139/687 [20%]
Flashes	382/687
Unilateral	347/382 [91%]
Bilateral	35/382 [9%]
Floaters	548/687
Unilateral	524/548 [96%]
Bilateral	24/548 [4%]
Acuity	
Acute (≤ 2 weeks)	653/687 [95%]
Sub-acute (> 2 and < 4 weeks)	20/687 [3%]
Chronic (≥ 4 weeks)	14/687 [2%]
Recent invasive ophthalmic intervention	
Yes	31/687 [5%]
No	656/687 [95%]
Directed to GES by healthcare provider	
Yes	79/687 [11%]
No	608/687 [89%]

### Consultation to OES

Consultation rate of OESs was 608/687 (89%) (Table 3). Multivariable logistic regression (Table 4) identified symptoms ≤ 2 weeks (OR 8.0; 95% CI 2.3–28; p = 0.001), ≥ 45 years age (OR 2.4; 95% CI 1.4–4.3; p = 0.002), UCC setting (OR 2.9; 95% CI 1.7-5.0; p < 0.001) as positively associated with consultation to OESs, and headache (OR 0.22; 95% CI 0.12–0.41; p < 0.001), and neurologic symptoms (OR 0.1; 95% CI 0.19–0.49; p = 0.005) as variables negatively associated with consultation to OESs. Recent invasive ophthalmic intervention, being directed to the GES by a healthcare provider, symptoms of decreased vision, and symptoms of visual field defect were not predictive of referral to OES in this model.

**Table 3:** Healthcare Utilization of Adult Patients Presenting with Flashes and/or Floaters to General Emergency Service Settings

Mean time from triage to discharge (hours ± SD)	2.43 ± 2.36
Total time triage to discharge (hours)	1,664
Mean cost of visit (CAD $ ± SD)	$139.11 ± $113.93
Total cost (CAD $)	$95,5570.54
Ophthalmology emergency services consulted	
Yes	608/687 [89%]
Total time triage to discharge (hours)	1,345
Total cost (CAD $)	$81,879.70
No	79/687 [11%]

**Table 4:** Multivariate Logistic Regression of Features Associated with Utilization of Ophthalmology Emergency Services for Adult Patients Presenting with Flashes and/or Floaters to General Emergency Services

Characteristic	Odds Ratio	95% Confidence Interval	P Value
Age ≥ 45 years	2.4	1.4–4.3	p = 0.002
Symptoms ≤ 2 weeks	8.0	2.3–28	p = 0.001
Urgent care setting	2.9	1.7–5.0	p < 0.001
Headache	0.22	0.12–0.41	p < 0.001
Neurologic symptoms	0.1	0.19–0.49	p = 0.005

*Recent invasive ophthalmic intervention, being directed to the GES by a healthcare provider, symptoms of decreased vision, and symptoms of visual field defect were not predictive of consultation of OES in this model.

### Healthcare utilization

The mean time spent in GESs for all F/F patients was 2.43 ± 2.36 h (range 0.15–30.5 h) from time of triage to discharge. In total this represented 1664 h (69 days) of patient occupancy in GESs (Table 3). The mean cost for each visit was $139.11 ± $113.93 and the total cost for all encounters to the hospital was $95,5570.54, all in Canadian dollars. In the subset of patients that ultimately received consultation from OESs, total wait time in GESs was 1345 h (56 days) and total cost of encounters to the hospital was $81,879.70.

## Interpretation

The negative consequences of overcrowding in GESs are considerable and abound in the literature[12]. Eye-related emergency visits contribute to this patient volume and research is needed around the health systems that currently exist to optimize patient care.

The epidemiology of eye-related complaints in GESs has been characterized in a variety of health care settings, representing 1.5–18% of all GES visits[4, 13–17], and has been increasing in volume over time[4]. In the United States, this represents approximately 2 million visits per year with an annual cost of 2 billion dollars[4]. Despite the incidence of eye-related visits to GES, several studies have highlighted the lack of comfort general emergency physicians have in managing eye-related issues.[18–20] Ophthalmology, along with orthopedics, represents one of the most frequently consulted services by GESs[16, 21].

Our current results demonstrate a consultation rate of 89%, for the population of adult patients presenting with F/F in GESs, appreciably higher than the 39.6% across all eye-related complaints in the ED reported by Wang et al.[22] at a tertiary care, university based medical center in the US, and the 20–40% overall consultation rate for all-comers to the ED[23]. However, these studies did not examine the nature of the presenting complaint, which would be the primary driver of consultation, as our study has.

The healthcare utilization of these patients ultimately referred to OESs is not insignificant. Roughly extrapolating based on population size, this would represent approximately 2 million dollars per year in hospital costs and over 31,845 h (1326 days) of patient waiting in GES setting within the province of Ontario per year.

Several proposals have been made to address the burden of emergency eye care[24, 25] including primary prevention of eye injuries, empowering general emergency physicians through medical education, equipment, and training, educating patients and local health care providers, and optimization of patient triage and so-called “front-end” operations, the last of which is most relevant to this study. Front-end processes are defined as the patient care processes that occur from the time of a patient’s initial arrival to a GES to the time a health care provider formally assumes responsibility, notably including the triage process[26]. Previous studies have demonstrated the efficacy of front-end operation targeted care pathways to improve the care of otolaryngologic and head and neck emergency patients[27], emergency presentations for cystitis[28], and common gynecologic emergencies[29]. In all studies, the identification of appropriate patients at triage for specifically designed care pathways resulted in a reduction in wait times and burden on health care providers without compromising safety.

While no study has specifically investigated triage directed expediated care pathways for eye-related complaints in GESs, Singman et al.[7] studied a related approach in implementing a center wide program allowing for same-day appointments in the ophthalmology outpatient clinic. Implementation of same-day appointments did not reduce the number of ED visits for ophthalmic complaints suggesting that the population utilizing same-day appointments is different than those presenting to the ED, or perhaps a masking of the effect of the diversion of ophthalmic complaints from the ED due to rising presentations to the ED in the post-intervention period for other reasons. Nevertheless, the study demonstrated significantly reduced costs associated with same-day appointments in the outpatient setting as well as significantly reduced transit time for the patients.

One challenge of symptom-oriented research and applying triage guided operations in GESs is the recognition of the lack of specificity of many symptoms, such as fatigue or weakness, to an isolated organ system or medical specialty[30]. Symptoms of F/F are relatively specific to the ocular system and occasionally the neurologic system, and can often further be refined to the posterior segment[10] of the eye with additional information from history available at time of triage. Another possible concern is the increased workload directed to the downstream service, in this case OESs. Presumably this would not be the case as the majority of these patients with F/F would eventually have OES consulted for them and the use of these pathways would simply identify these patients earlier, in fact leading to a decreased overall medical workload. A frequently voiced concern of bypassing evaluation in the ED/UCC is that of significant pathology being missed. However, a review of ED-based strategies to divert patients to their primary care provider or a designated primary care clinic found diversion strategies to be no less safe or more harmful than standard ED care. An important distinction is that these studies investigated diversion as opposed to facilitation from the ED[31]. The consultation rate of 89% in our population of F/F patients reflects only a rudimentary criteria examining purely symptoms of F/F. Conceivably, any attempt at selecting patients for an expediated care pathway would adopt a more nuanced approach. For example, setting further criteria based on only 3 additional factors identified by our multivariable regression results and examining the population of patients that were ≥ 45, had acute symptoms, and lacked headache and neurologic symptoms, reveals that 94% (468/499) of patients had OESs consulted. Using other additional features such as basic vital signs other systemic symptoms could help further select outpatients that should proceed through GESs rather than an expediated care pathway. These statistically notable factors reflect the clinical framework physicians would use in determining whether to consult OESs. Patients with symptoms of headache and neurologic may be more likely to have non-ocular diagnoses such as migraine or stroke. Posterior vitreous detachment is a process related to aging and those < 45 are less likely to require an examination of sequelae of posterior vitreous detachment with OESs. Presentation to UCC is a unique feature to the local health system where the university eye clinic is physically in the same building as the SJHH UCC. Patients with previous ocular history that would have been managed at the eye clinic would be more likely to present to the associated UCC and emergency physicians may be more inclined to consult for a patient because of the physical proximity. In general, patients with F/F may regard their condition as less acute than requiring presenting to the ED and favor presenting to the UCCs.

### Limitations

Several limitations exist within the current study. Triage chief complaint data was collected retrospectively and thus subject to the inherent bias and variability of the patient relaying subjective information and the nurse or physician’s documentation of the communication. In addition, several features that are known to be associated with the presence of retinal tear/detachments and might increase the likelihood of consultation of OES such as high myopia or history of previous retinal tear/detachment could not be determined from triage text. Nevertheless, this triage data has certain strengths by reflecting current real-life practices in GESs. In addition, there is a degree of subjectivity in the translation of the free text of the triage complaint into specific symptoms. To mitigate this, in the creation of the database a single author (CS), blinded to the status of ophthalmology consultation, coded all symptoms from triage free text using standardized keyword terms. However, a prospective, standardized method of collecting triage information and symptom coding from patients could be performed in the future to achieve improved consistency in this regard. This study was conducted in a setting of a publicly funded health care system in a tertiary, university associated medical center and the results may not be directly applicable to other models of providing emergency eye care. While variations in local healthcare systems may exist in the delivery of the emergency eye-care for these patients, the natural flow from general emergency service to an eye care provider that can provide a dilated fundus exam is consistent due to the nature of the complaint, and the concept of reducing unnecessary waiting and streamlining appropriate care is generalizable in any setting where patients with F/F are making first contact to the healthcare system in GESs.

## Conclusion

Due to a multitude of factors, a high proportion of patients presenting with flashes and/or floaters in general emergency service settings require consultation by ophthalmology emergency services. In the current framework of care, these patients can contribute to emergency service volume, consume healthcare resources, and spend significant time waiting before their contact with an eye care provider. Ultimately, we hope this health systems research will help inform future practice patterns when it comes to the triage of eye-related complaints by contributing to the knowledge needed to guide prospective studies on innovative care pathways appropriate for local health systems.

## Electronic supplementary material

Below is the link to the electronic supplementary material.

Supplementary Material 1

## Data Availability

The datasets generated and/or analysed during the current study are not publicly available due patient privacy but are available from the corresponding author on reasonable request.
