# Supplementary material for: The burden of flashes and floaters in traditional general emergency services and utilization of ophthalmology on-call consultation: a cross-sectional study
Source: BMC Ophthalmol. 2022 Oct 4;22:394. doi: 10.1186/s12886-022-02613-6 (PMC9530426; doi:10.1186/s12886-022-02613-6)
Supplement: Supplementary file 1 — Supplementary Material 1 [file 12886_2022_2613_MOESM1_ESM.docx]

**Supplemental Appendix**Criteria for triage text coding to dichotomous symptom variables

Selected symptom coding schema:

[symptom]_[L]

Where L = U if unilateral, L = B if bilateral, or _[L] = null if systemic symptom

Flashes: flashes, lightning, lights, sparks, fireworks, photopsia, flickering, (not better represented by another symptom)

Floaters: floaters, dots, spots, lines, squiggles, specks, cobwebs, strings, blobs, particles, streaks, flecks, floating “object(s)”, snow(globe), localized film or veil, (not better represented by another symptom)

Headache: headache, migraine, pressure/pain described specifically to the head, (not better represented by another symptom)

Neurologic: paresthesia, numbness, seizures, unilateral weakness, gait disturbance, tremor, speech difficulty, memory change, confusion, disorientation, (not better represented by another symptom

Example triage text to coding conversion:
Visual Disturbance (rt lightning flashes lower rt corner of eye. large floaters. flashes on monday. headache all week)

Flashes_u;floaters_u;headache
